# Care of pregnant women with pre-existing medical conditions in German perinatal centers

**DOI:** 10.1007/s00404-025-08016-4

**Published:** 2025-04-07

**Authors:** P. Kosian, B. Strizek, S. Kehl, M. Abou-Dakn, E. Jost, W. M. Merz

**Affiliations:** 1 Department of Obstetrics and Prenatal Medicine, University Hospital Bonn, Bonn, Germany; 2 Department of Obstetrics and Gynecology, LMU University Hospital, LMU Munich, Munich, Germany; 3 Department of Obstetrics and Gynecology, St Joseph Hospital, Berlin, Germany

**Keywords:** Pre-existing medical condition, Pregnancy, Perinatal care, Germany, Surveys and questionnaires

## Abstract

**Introduction:**

Pregnancies in women with chronic medical conditions are characterized by a higher maternal and perinatal complication rate during pregnancy, childbirth, and the postpartum period. The German Maternity Guideline does not provide specific recommendations for the care of these women. The aim of this study was to evaluate the care of pregnant women with pre-existing medical conditions in German perinatal centers (Level 1 and 2) and perinatal care level 3 hospitals.

**Materials and methods:**

Based on guidelines and literature, seven topics were identified: preconception counseling, timing of consultation, care for pregnant women with rare diseases, participation in continuing education, multidisciplinary case conferences, resources for patient counseling, and transfer of the patient to another center. Representatives of all perinatal centers were contacted by email and invited to participate. The anonymous online survey was conducted using the SoSci Survey platform.

**Results:**

Of 310 centers, 103 (33.2%) representatives responded. 62.2% (*n* = 64) reported managing 11–30 pregnant women with pre-existing conditions per month. 22.1% (*n* = 23) of all centers regularly care for pregnant women with rare diseases, and 46.6% offer preconception counseling. University hospitals offer these services more frequently. Regular case conferences are held in 34.0% of centers, and 80.6% of medical staff regularly participate in continuing education on the topic.

**Conclusion:**

According to the results of our survey, 76.7% (*n* = 79) of perinatal centers regularly care for patients with pre-existing conditions, while only 22.1% care for patients with rare diseases. The findings highlight the need to implement standardized recommendations and targeted resource allocation to ensure optimal care for this patient group.

**Supplementary Information:**

The online version contains supplementary material available at 10.1007/s00404-025-08016-4.

## What does this study add to the clinical work

**Table:** 

This study is the first to investigate current practices of management for pregnant women with pre-existing medical conditions across perinatal centers in Germany. Implementation of standardized training in"Obstetric Medicine"as already established in anglophone countries [17] and structured preconception counseling and co-care according to the pre-existing medical condition could be important next steps in further improving care for this group of patients.	

## Introduction

The proportion of pregnant women with pre-existing medical conditions has tripled since the beginning of the millennium and varies depending on the region investigated. A population-based study by Lundborg et al. calculated the prevalence of at least one pre-existing medical condition 5 years prior to childbirth in Sweden with 8.7%, a threefold increase between 2002 and 2019 [1]. Data from British Columbia, Canada, show a prevalence of 26.2% within 5 years before childbirth [2]. Pregnancy, childbirth, and the postpartum period in these women are characterized by an increased rate of maternal and perinatal complications [3]. In recent years, the proportion of multimorbid pregnant women (defined as ≥ 2 chronic pre-existing medical conditions) has increased and a dose-dependent association between the number of co-existing chronic medical conditions and the likelihood of adverse maternal outcomes, such as severe maternal morbidity or mortality [3–5], has been revealed. Relevant maternal complications (e.g., acute kidney failure, sickle cell crisis, heart failure) during pregnancy, childbirth, and the postpartum period are categorized as severe maternal morbidity (SMM) [6] and can lead to significant short- or long-term consequences for the mother [7]. Moreover, the occurrence of SMM negatively impacts perinatal outcome, increasing the risk of a 5-min Apgar score < 7, admission to neonatal intensive care unit (NICU), and perinatal and neonatal mortality [8]. SMM result in substantial costs for healthcare systems and society due to associated maternal and perinatal complications [9, 10].

Care of high-risk pregnancies in Germany is provided by office-based gynecologists, other medical specialists for pre-existing medical conditions, and perinatal centers. A risk catalog including obstetric risk factors and maternal pre-existing medical conditions exists, but specific guidelines for the antenatal care of pregnant women with pre-existing medical conditions cannot be derived from the German Maternity Guideline [11]. Multidisciplinary co-management is not regulated or mandatory [11]. To date, the management of care for this patient group at German perinatal centers has not been investigated. Therefore, the aim of this survey was to assess antenatal care of pregnant women with pre-existing medical conditions at German perinatal centers (Level 1 and 2) and perinatal care level 3 hospitals (Level 3).

## Methods

Based on the literature, the following topics were included in the survey: preconception counseling and timing of referral for co-management [12, 13], care for pregnant women with rare diseases (defines as prevalence ≤ 5/10,000 individuals), resources for patient counseling (clinical decision support systems (CDS systems) such as online databases, interdisciplinary consultations, guidelines, PubMed, online teratology information services (Embryotox) [14] and textbooks), the necessity of transferring patients to another center in cases of maternal complications related to underlying conditions, continuing education, and the organization of multidisciplinary case conferences [5]. A total of 11 questions were developed (Supplemental File).

Representatives of all 310 perinatal centers (Level 1, Level 2) and perinatal care level 3 hospitals were invited to participate in the survey via email in January 2024. Contact details were obtained from the list of perinatal centers available at www.perinatalzentren.org. Care levels are categorized as follows: Level 1: estimated birth weight of less than 1250 g or gestational age of less than 29 + 0 weeks. Level 2: estimated birth weight between 1250 and 1499 g and at least 32 + 0 weeks of gestational age. Level 3: estimated birth weight of at least 1500 g.

Two reminders were sent at 4-week intervals, and the survey was concluded in March 2024.

The anonymous online survey was conducted using the SoSci Survey platform [15]. The SoSci Survey online questionnaire was made available to participants at www.soscisurvey.de [16]. Data analysis was performed using IBM SPSS Version 27 (SPSS Inc., Chicago, IL, USA). In addition to descriptive statistics, subgroup differences were analyzed using Pearson’s Chi-square test. A *p* < 0.05 was considered statistically significant.

## Results

**Fig. 1:**
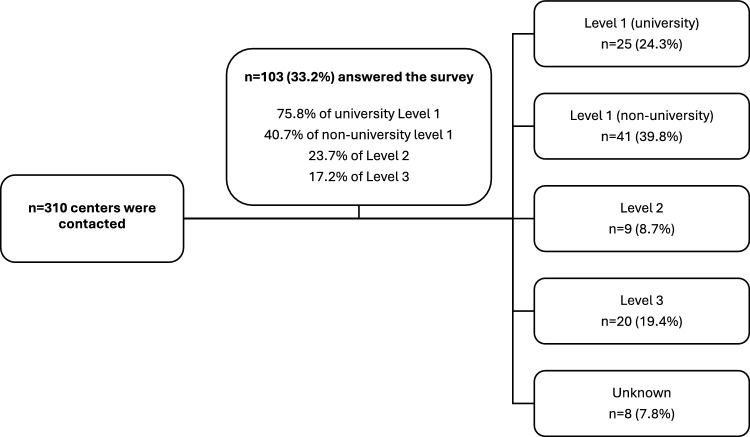
Flow chart of contacted and participating perinatal centers. Proportion of Level 1 (university), Level 1 (non-university), Level 2, and 3 centers

In total, 33.2% (*n* = 103) completed questionnaires were available for analysis (Fig. 1). The majority (62.2%; *n* = 64) reported managing 11–30 pregnant women with pre-existing medical conditions per month, while 15 centers treated more than 30 patients per month (Table 1).

**Table 1:** Number of pregnant women with pre-existing medical conditions presenting monthly at German perinatal centers (Level 1, 2 and 3). *n* = 9 (8.7%) of the representatives did not provide an answer

Center type	0–10	11–20	21–30	> 30
Level 1 (university)	1	5	13	5
Level 1 (non-university)	6	14	11	10
Level 2	1	5	3	0
Level 3	7	8	5	0
**Total number *n* (%)**	15 (14.6)	32 (31.1)	32 (31.1)	15 (14.5)

Bold values represents the sums of all the centers in the different groups taking care of different numbers of patients

70.9% (*n* = 73) indicated establishment of a dedicated special consultation service. The majority of hospital representatives was familiar with the category of rare diseases (96.1%; *n* = 100), and 22.1% (*n* = 23) of all centers regularly provide care for this subgroup of patients. More frequently this is established at university hospitals (60.0% vs. 8.6%; *p* = 0.001).

**Fig. 2:**
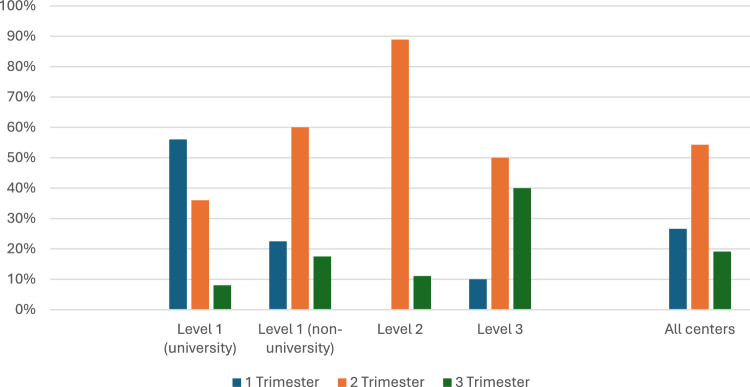
Timing of initial consultation at perinatal centers (Level 1 (university), Level 1 (non-university), Level 2, Level 3 and all centers) in pregnant women with pre-existing medical conditions; *p* = 0.001

Preconception counseling is provided by 46.6% of centers, more often at university hospitals (88.0% vs. 33.3%; *p* = 0.001). Timing of initial consultation at perinatal centers for pregnant women with pre-existing medical conditions is found in Fig. 2. The majority (53.4%; *n* = 55) recommend initial consultation in second trimester, whereas 26.6% (*n* = 27) advocate co-management in a perinatal center starting in first trimester (Fig. 2).

Regular case conferences are held in 34.0% of centers.

A total of 56.3% (*n* = 58) offer in-house continuing education, and 80.6% (*n* = 83) reported that medical staff regularly participates in external continuing education on the topic.

**Fig. 3:**
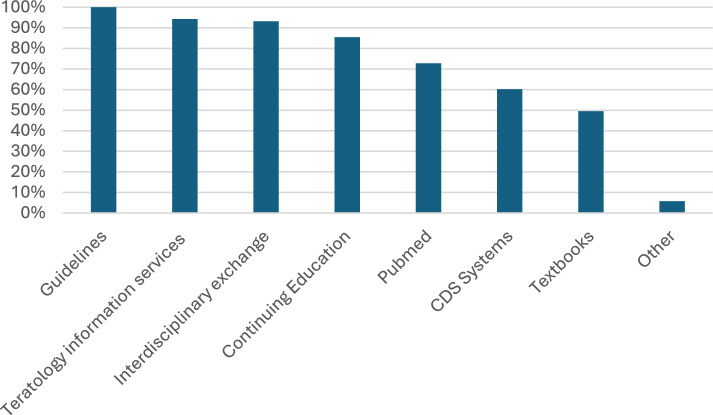
Resources used to care for pregnant women with pre-existing medical conditions in perinatal centers. Own statement (Other): University hospital inquiry and own research. Multiple answers were possible

The full range of resources utilized for counseling pregnant women with pre-existing medical conditions is found in Fig. 3.

Representatives were asked about the necessity of inter-hospital transfer. 24% of university Level 1 centers, compared to 56.1% of non-university Level 1 centers and 100% of Level 2 and 3 centers, reported the occasional need for transferring patients in cases of maternal complications related to underlying conditions (*p* = 0.001).

## Discussion

In the United Kingdom, in addition to establishing a subspecialty in “Obstetric Medicine” dedicated to the care of pregnant women with chronic medical conditions, a model also adopted in other anglophone countries such as the United States of America, Canada, Australia, and New Zealand [17]—the “Maternal Medicine Network” was launched in 2021. This network categorizes pre-existing medical conditions based on their relevance for pregnancy into three levels, corresponding to recommended degrees of co-management at specialized centers as follows: (1) local routine obstetric care, (2) consultation with a specialized center, and (3) regular co-management at a specialized center [18]. In Germany, the Maternity Guideline does not include any specific requirements, or recommendations for timing of initial consultation in perinatal centers for pregnancies complicated by pre-existing medical conditions [11].

In our survey, 46.6% of centers reported to provide preconception counseling; however, it remains unclear how many patients with pre-existing medical conditions in Germany are referred for preconception counseling overall and how many of these make use of it. A systematic review by Nana et al. [19] suggests that preconception counseling for patients with pre-existing medical conditions can improve perinatal and maternal outcome, thereby positively impacting the course of pregnancy [19]. Further, Dude et al. demonstrated that routine examinations for women with chronic medical conditions in the USA 1 year before conception are associated with decreased risk of maternal morbidity and mortality [20]. German guidelines recommend preconception counseling for certain pre-existing medical conditions such as congenital heart disease, kidney disease, systemic lupus erythematosus, and antiphospholipid syndrome [21–23]. However, structured implementation, funding, and establishment within the German healthcare system has not yet been achieved.

Our survey revealed that university Level 1 centers (56%), compared to non-university level 1 (36%) Level 2 (0%) and 3 centers (10%), significantly more often schedule pregnant women with pre-existing medical conditions for consultation in first trimester. Whether this is related to differences in patients’ medical conditions or due to organizational factors cannot be deducted from our study. It is likely that university hospitals more frequently care for pregnant women with complex pre-existing medical conditions (e.g., cardiac or renal diseases) which necessitate multidisciplinary, centralized co-management starting as early as first trimester [24].

For the majority, care of pregnant women with rare diseases is performed at university hospitals. This may be attributed to the foundation of the “National Action League for People with Rare Diseases” (NAMSE) in 2011 which includes the establishment of centers for rare diseases and to the complexity of these conditions often requiring co-management at specialized centers. Despite these improvements, patients continue to face challenges [25], which can further aggravate during pregnancy.

While all representatives who answered our questionnaire consult guidelines for counseling their patients, only 60.2% use CDS systems like online databases, the most commonly known being “Uptodate” [26]. A systematic review by Gholamzadeh et al. [27] investigated the use of CDS systems in the care of patients with chronic diseases. It was shown that consultation of CDS systems has a positive impact on clinical decision-making processes and can improve care and treatment of patients [27]. In addition to guidelines, current study findings, multidisciplinary care, and personal experience, CDS systems, therefore, may also play an important role in the care of pregnant women with pre-existing medical conditions.

Despite reporting regular care for patients with pre-existing medical conditions, all Level 2 centers and Level 3 hospitals indicated that they occasionally transfer patients to other facilities due to complications of the underlying condition. Likewise, 24% of university hospitals reported the necessity to occasionally transfer patients to other centers. Since these are tertiary care centers, it can be speculated either that the question was possibly misunderstood and interpreted as transfer to another department within the hospital or that there is misallocation of patients even in university Level 1 centers.

A multidisciplinary approach for care of pregnant women with pre-existing medical conditions is essential to achieve the best possible maternal and perinatal outcome [28]. In our survey, 93.2% of centers reported that they would provide multidisciplinary care for their patients if necessary.

The decision-making process by office-based gynecologists regarding referral for co-management and whether pre-existing medical conditions themselves play a role in this process remains unknown. Therefore, an important next step is to conduct a survey on this topic among office-based gynecologists in Germany. Further, implementation of standardized training in “Obstetric Medicine” as already established in anglophone countries [17] and structured preconception counseling and co-care according to the pre-existing medical condition could be important next steps in further improving care for this group of patients.

Limitation of the study is the comparatively low number of level 2 and 3 centers participating in the study as well as the expected overall low response rate of 33%. Whether care for woman with pre-existing medical conditions is provided at the majority of non-responding centers remains an open question.

## Conclusion

In conclusion, this study is the first to investigate current practices of management for pregnant women with pre-existing medical conditions across perinatal centers in Germany. A substantial proportion of centers have established dedicated consultation services, and university centers demonstrate higher engagement in specialized care, including regular care for pregnant woman with rare diseases, preconception counseling, and early co-management during first trimester. However, our survey revealed considerable differences according to the level of perinatal centers and underline lack of standardized protocols and resource allocation to ensure effective care for this patient population.

## Supplementary Information

Below is the link to the electronic supplementary material.

Supplementary file1 (PDF 32 KB)

## Data Availability

No datasets were generated or analysed during the current study.
