# Supplementary material for: Care of pregnant women with pre-existing medical conditions in German perinatal centers
Source: Arch Gynecol Obstet. 2025 Apr 7;312(1):267–72. doi: 10.1007/s00404-025-08016-4 (PMC12177008; doi:10.1007/s00404-025-08016-4)
Supplement: Supplementary file 1 — Supplementary file1 (PDF 32 KB) [file 404_2025_8016_MOESM1_ESM.pdf]

Sehr geehrte Kolleginnen und Kollegen,

in den letzten Jahren ist eine stetige Zunahme von Schwangerschaften bei Frauen mit chronischen Erkrankungen zu verzeichnen. Im Rahmen dieser kurzen Umfrage soll mit Ihrer Hilfe und der Unterstützung der Sektion "Maternale Erkrankungen" der DGGG die Versorgungsstruktur der Betreuung Schwangerer mit Vorerkrankungen in deutschen Perinatalzentren erhoben werden.
Hierbei möchten wir uns ausschließlich auf Vorerkrankungen, die für die Schwangerschaft relevant erscheinen, fokussieren. Eine unkomplizierte substituierte Hypothyreose oder ein Gerinnungspolymorphismus (wie z.B. Faktor V Leiden heterozygot) ohne thromboembolische Ereignisse in der Eigenanamnese sind hiermit nicht gemeint. Vielmehr verstehen wir für den Zweck dieser Umfrage darunter schwerwiegendere Vorerkrankungen wie z.B. eine Multiple Sklerose oder chronisch entzündliche Darmerkrankungen.

Vielen Dank, dass Sie sich kurz die Zeit nehmen (ca. 2min., 11 Fragen) um folgenden Fragebogen anonym zu beantworten.

1) Welches Level ist Ihr Perinatalzentrum?

- Level 1 (Universitätsklinikum)
- Level 1 (nicht-universitär)
- Level 2
- Perinataler Schwerpunkt

2) Wie viele Patientinnen stellen sich ungefähr monatlich zur Mitbetreuung bei einer für die Schwangerschaft relevanten Vorerkrankung in Ihrer Klinik vor?

- 0-10
- 11-20
- 21-30
- Eigene Angabe:

3) Gibt es eine Spezialsprechstunde/Risikosprechstunde für die Betreuung dieser Patientinnen?

- ja
- nein
- Falls ja, hat Ihre Sprechstunde einen besonderen Schwerpunkt? Details:

4) In welchem Trimenon empfehlen Sie bei Schwangeren mit Vorerkrankungen die Erstvorstellung in Ihrer Klinik?

- 1. Trimenon
- 2. Trimenon
- 3. Trimenon

5) Gibt es interdisziplinäre Fallkonferenzen zur Besprechung dieser Patientinnen?

- ja, regelmäßig
- ja, bei Bedarf
- nein

Abteilung für Geburtshilfe und Pränatalmedizin
Sektion Maternale Medizin
Zentrum für Geburtshilfe und Frauenheilkunde
Universitätsklinikum Bonn
Venusberg Campus 1, 53127 Bonn
Ansprechpartner: Dr. med. Philipp Kosian, Prof. Dr. med. Waltraut Merz
Email: philipp.kosian@ukbonn.de; Tel.: 0228/287/40154

6) Welche der folgenden Ressourcen werden für die Beratungen von Patientinnen mit einer für die Schwangerschaft relevanten Vorerkrankung in Ihrer Klinik herangezogen. Bitte alle zutreffenden Punkte ankreuzen.

- Online-Plattformen (z.B. UptoDate, Amboss)
- Eigene klinische Erfahrung und Austausch mit anderen involvierten Fachrichtungen
- Leitlinien
- Literatursuche auf PubMed
- Embryotox
- Monografien/Lehrbücher zur Thematik (z.B. Briggs Drugs in Pregnancy and Lactation)
- Fortbildungen/Kongresse
- Sonstiges (Details):

7) Kommt es vor, dass Patientinnen aufgrund von Komplikationen der Grunderkrankung prä- oder postpartal in ein anderes Zentrum verlegt werden müssen?

- ja
- nein

8) Bieten Sie eine präkonzeptionelle Beratung für Patientinnen mit Vorerkrankungen an?

- ja
- nein

9) Haben Sie Erfahrung mit der Betreuung Schwangerer mit seltenen Erkrankungen (Orphanet Diagnosen)? Bitte Zutreffendes Ankreuzen. Mehrfachantworten möglich.

- ja, regelmäßig
- ja, vereinzelt
- nein
- Ich habe mich mit dem Thema seltene Erkrankung in Kombination mit Schwangerschaft bis dato noch nicht ausgiebig beschäftigt
- Ich kannte die Subgruppe der seltenen Erkrankungen bis dato nicht

10) Gibt es ein klinikinternes Fortbildungsangebot zum Thema Vorerkrankung und Schwangerschaft?

- ja
- nein

11) Nehmen ärztliche KollegInnen regelmäßig an Fortbildungen zum Thema Schwangerschaft und Vorerkrankung teil?

- ja
- nein

Abteilung für Geburtshilfe und Pränatalmedizin
Sektion Maternale Medizin
Zentrum für Geburtshilfe und Frauenheilkunde
Universitätsklinikum Bonn
Venusberg Campus 1, 53127 Bonn
Ansprechpartner: Dr. med. Philipp Kosian, Prof. Dr. med. Waltraut Merz
Email: philipp.kosian@ukbonn.de; Tel.: 0228/287/40154
